# A Novel Approach for Tissue Analysis in Joint Infections Using the Scattered Light Integrating Collector (SLIC)

**DOI:** 10.3390/bios15120795

**Published:** 2025-12-04

**Authors:** Elio Assaf, Cosmea F. Amerschläger, Vincent B. Nessler, Kani Ali, Robert Ossendorff, Max Jaenisch, Andreas C. Strauss, Christof Burger, Gunnar T. Hischebeth, Phillip J. Walmsley, Dieter C. Wirtz, Robert J. H. Hammond, Damien Bertheloot, Frank A. Schildberg

**Affiliations:** 1 Department of Orthopedics and Trauma Surgery, University Hospital Bonn, 53127 Bonn, Germany; 2 Institute for Medical Microbiology, Immunology and Parasitology, University Hospital Bonn, 53127 Bonn, Germany; 3 School of Medicine, University of St Andrews, St Andrews KY16 9TF, UK

**Keywords:** bacteria, detection, diagnosis, periprosthetic joint infections, septic arthritis, musculoskeletal infections

## Abstract

Total joint arthroplasty is among the most common surgical procedures performed worldwide, with frequency increasing due to demographic changes. Accelerating the diagnostic process using new techniques is crucial for effective therapy. This pilot study aims to test such innovative technology in the context of periprosthetic joint infection (PJI) using Scattered Light Integrating Collector (SLIC) technology. While we wish to evaluate whether SLIC can be used to reliably detect the status of infection within human tissue samples in the future, our current research focused on building its foundation by evaluating steps of sample preparation that allow for heightened growth depiction. It is, to our knowledge, the first study concerning the usage of solid human tissue samples using the SLIC device. Adult patients presenting with native or periprosthetic joint infections were included in this prospective study. Biopsies were obtained using sequential sampling, and bacterial density was optimized through titration series. Cryopreservation and agents influencing coagulation were investigated. Our study demonstrates that simple pretreatment could aid in detecting pathogen growth in infected tissue samples. Findings showed a clear advantage for no addition of agents affecting coagulation. Additionally, our protocols proved reliable after prolonged cryopreservation at −20 °C for up to 8 weeks, showing no significant difference compared to primary testing. AUC comparison showed comparable results for sample storage at −80 °C for up to 8 weeks. Similar outcomes were seen for samples ranging from 25 µL to 300 µL, with biological replicates displaying higher thresholds for larger volumes without significant differences. This study introduces a simple and quick diagnostic tool for detecting bacterial growth using tissue biopsies and develops an SOP for further research with this innovative technique. The suggested SOP enables SLIC to hint at an underlying bacterial infection within 5 h using joint tissue, offering a possible novel approach in diagnosing periprosthetic joint infections and septic arthritis. While not yet designed to compare sensitivity to other culture methods, it provides a solid basis for further clinical research.

## 1. Introduction

Total joint arthroplasty is a common operation worldwide, with infection rates between 1% and 3% [1,2]. With an aging population, there has been a notable rise in the primary implantation of hip and knee joint endoprostheses both in Germany and globally, with further increases anticipated in the future [2,3,4]. This development is linked to a substantial increase in prosthetic infections, presenting a significant challenge for healthcare providers, patients, and their social networks [2,5].

Prosthetic joint infections (PJIs) result in extended and repeated hospital stays [5,6]. Aside from a decline in quality of life and mobility, patients also experience a significantly higher mortality rate [2,7]. The mortality rate of hip prosthesis infections is 13.6% at one year and 25.9% at 5 years, which is comparable to the mortality rate of several common tumors [5].

In addition to prosthetic joint infections, septic arthritis (SA) of native (nonprosthetic) joints is also a significant concern, affecting 2 to 6 individuals per 100,000 persons per year [8]. Although generally monoarticular, it can also occur polyarticular [9]. Approximately 50% of SA cases in adults involve the knee, followed by other large joints of the skeleton, including hip, ankle, elbow, wrist, and shoulder [9,10].

When there is suspicion of PJI, a severe complication after total joint arthroplasty, identifying the causative microorganisms can be a challenge [6,7,11]. An estimated 7–12% of patients show negative cultures despite clear clinical signs of infection, with administration of antibiotics in the weeks before culture collection frequently being the reason for this [8,11].

Severe infections and the potential for them to result in bacteremia and sepsis require rapid and effective pathogen identification [1,10]. In addition to surgical treatment, an adequate antibiotic therapy, usually over 12 weeks, is necessary [11,12]. Rapid information about pathogen identity and antibiotic effectiveness is crucial, especially at a time when the number of infections caused by multi-resistant bacteria has increased worldwide [1,12,13]. The detection and characterization of small amounts of bacteria is difficult because bacterial culture and antibiotic resistance studies often take 24 h or longer [14].

Against this background, a new technique, the “Scattered Light Integrating Collector (SLIC)” technique, was developed. The method is intended to reduce the detection limit and the time to positive detection of the growth of small inocula [15]. Previous studies have already proven its detection limit to be between 10 and 100 colony-forming units (CFU) per mL in a volume of 1 mL using bacterial cultures [1,14,15].

There is no single routine test available that can accurately diagnose PJI, as each method has its pros and cons. While joint aspiration remains the most common and widely used method for diagnosing joint infections, tissue sample analysis has proven to be more effective in detecting co-infections. Additionally, sonicate fluid culture serves as a supplementary diagnostic tool for detecting PJI. The best results are achieved by combining multiple techniques [1,6,16,17,18].

Recent studies using SLIC focused on analyzing bacterial cultures. To our knowledge, SLIC has not been tested on tissue samples in the context of PJI [15]. Enhancing the detection of infectious agents can expedite diagnosis, enabling the timely initiation of appropriate treatment strategies. This is crucial in severe cases where rapid intervention can significantly improve patient prognosis [1,10].

This pilot study aims to identify sample treatment methods that can expedite and increase bacterial growth monitoring using SLIC in infected tissue samples derived from revision surgeries. It establishes the first standard operating procedure for this technique, facilitating and standardizing potential further research while possibly easing the hardship of PJI diagnosis as a cost-efficient additional tool in the future.

## 2. Materials and Methods

### 2.1. Sample Collection

We included patients with a clinical suspicion of joint infection and positive pathogen detection using microbiological tests suggested by the European Bone and Joint Infection Society (EBJIS) [19]. Tissue samples of the same patient and revision operation had to show a positive microbiology culture result within the microbiology department of the University Hospital Bonn to be included in this analysis. Written consent was obtained from each patient. Samples were transported from the operating room (OR) to the laboratories of the orthopedics and traumatology department at the University Hospital Bonn. If immediate analysis was not feasible, samples were stored at 4 °C for up to 24 h after collection [20]. The study was authorized by the institutional ethical committee of the University Hospital Bonn (ID: 110/23-EP).

### 2.2. Sample Processing

A combination of vortexing and other techniques, like sonication or cell culture, plays a major role in the diagnosis of PJI, showing higher detection levels than sonication alone [1,21,22]. Therefore, following microbiology procedures for tissue biopsy testing, we decided to store the tissue in sterile containers with 5 mL of sterile saline solution and 2 mm sterile steel balls [1]. This setup facilitates the extraction of bacteria from infected tissue into the saline solution [23]. Aligned with sonication conditions, samples were placed in sterile saline solution and disrupted using vortexing and sterile steel balls for increased impact. Initial vortexing was performed for 60 s before the disrupted tissue and fluid were separated using a 40 µm cell-strainer (Greiner Bio-one GmbH, Kremsmünster, Austria). Müller-Hinton Broth (MHB; Carl Roth GmbH, Karlsruhe, Germany), preheated at 37 °C, was added in a 1:5 ratio, followed by 10 s of vortexing to homogenize the contained bacteria and surrounding fluids.

**Figure 1:**
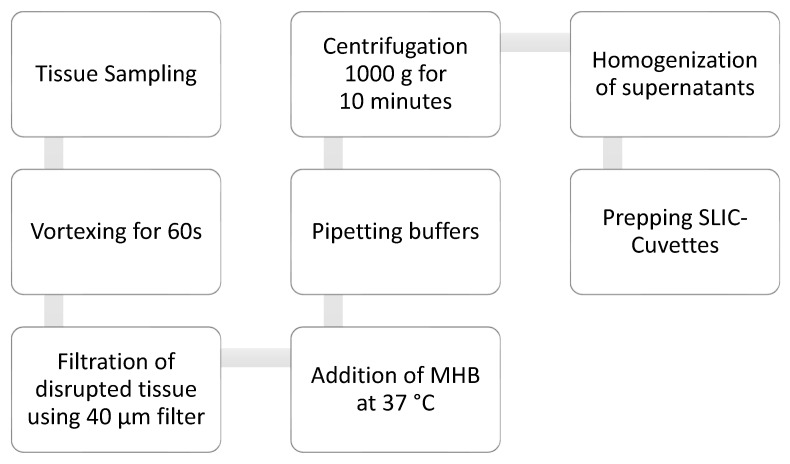
Workflow of sample processing.

Samples underwent centrifugation for 10 min at a standardized speed of 1000× *g*. Resulting supernatants were separated from pellets and homogenized individually using a vortex. For preparations of the SLIC cuvettes (BRAND GmbH + Co KG, Wertheim am Main, Germany), a definitive content volume of 1.5 mL was set. Cuvettes were filled with 1.4 mL MHB and 100 µL of the centrifuged sample. Additionally, 1.5 mL of MHB was used as a negative control (=blank). Analysis was run using SLIC Version 7.1 for 5 h. The workflow of the sample processing is illustrated in Figure 1.

#### 2.2.1. Coagulation Interference Tests

Before centrifugation, 1.5 mL of the fluid sample was pipetted into three different tubes (S-Monovette^®^/Sarstedt-Monovette Systems, Nümbrecht, Germany) with pre-added agents influencing coagulation. Silicate was used as a procoagulant. EDTA and heparin were chosen as anticoagulants. A plain tube without any additives (Greiner Bio-one GmbH, Kremsmünster, Austria) served as a control buffer. After centrifugation, supernatants were homogenized separately as previously stated. Four cuvettes were prepared with 1.4 mL of preheated MHB (37 °C) and 100 µL of sample from each tube. A blank was prepared using a native medium. The experimental runtime was set to 5 h (300 min).

#### 2.2.2. Storage Tests

To test different storage conditions, processed samples were allocated into cryotubes, each containing 150 µL. A set for each planned SLIC run was stored for 1 week at 4 °C, 2 weeks at −20 °C/−80 °C, 4 weeks at −20 °C/−80 °C, and 8 weeks at −20 °C/−80 °C, respectively. For storage testing, samples were thawed at room temperature and then vortexed for homogenization. Cuvettes were prepared with 1.5 mL of MHB (37 °C) and 100 µL of thawed tissue sample. A control was prepared as described above. The duration of the SLIC analysis was set at 5 h.

#### 2.2.3. Sample Amount

For titrations, all samples were prepared using Lithium-Heparin S-Monovettes^®^ (Sarstedt). Sample preparation was followed according to the previously mentioned procedure. A final extraction of various sample volumes followed and was distributed into 5 cuvettes with a total volume of 1.5 mL in each cuvette. Sample amount was distributed as follows: 25 µL, 50 µL, 100 µL, 200 µL, and 300 µL. The remaining volume to reach the final cuvette level of 1.5 mL was filled with MHB at 37 °C. Experiments were run for 5 h.

### 2.3. Statistical Analysis

SLIC output was extracted as a csv file and further interpreted using a previously programmed RStudio file (R version 4.3.1 (16 June 2023), 2023, The R Foundation for Statistical Computing, Vienna, Austria). This file exports different parameters of analysis (see below) as singular Excel files and plots the measured scattering as graphs to depict growth.

Scattering of laser light is measured each second and set in difference to the initial data depicting the gradient of the curve. The output signal value is originally measured in dB and converted into mV using R. Excel files (Microsoft^®^ Excel for Mac, version 16.89.1) were created listing measured values such as AUC, slope, and curve endpoint. Experiments were repeated at least 3 times, and data are represented as average ± SEM of 3 independent experiments. Statistical analysis and data visualization were performed using GraphPad Prism (version 10.2.3 for Mac, GraphPad Software, Boston, MA, USA, www.graphpad.com). The D’Agostino-Pearson test or graphical analysis was performed to assess normality in all measured values.

#### 2.3.1. Area Under the Curve (AUC)

AUC was calculated within an RStudio file (see above) using the trapezoidal function for determining the individual curve surface. Results were compared to the AUC of a constant value, which was set to the standard deviation of the specific curve at 2 min. When that surface area is surpassed, AUC methods consider the curve as growth.

#### 2.3.2. Endpoint Analysis (Mean 300 min)

The Mean 300 analysis represents the endpoint reached by the curves after a five-hour SLIC run. The mean is calculated in RStudio using 60 SLIC measurements taken every second during the final minute (the 300th min).

#### 2.3.3. Slope

Slopes are calculated individually using RStudio for each measured minute of a curve within an experiment between the curve origin and the evaluated minute. These data sets are then used to determine a mean slope for each curve over the 5 h timeframe. Comparison with the equivalent slope of the associated blank curve is performed using an unpaired *t*-test.

#### 2.3.4. Data Folds (Storage Testing)

For storage tests, the reproducibility of corresponding curves is assessed using the ratio between values of the storage test and the original (fresh) test. AUC, endpoint, and mean slope analyses are all used separately for this method. Ideally, values within one method would be identical, resulting in ratios equal to 1. Ratios were tested against this ideal fold using a *t*-test. Insignificant deviation confirms reproducibility.

### 2.4. SLIC Technology

The Scattered Light Integrated Collector (SLIC) uses a laser current directed through a liquid sample. It is scattered after hitting the contained bacteria. As bacterial cells propagate, so does the amount of scattered laser signal, thereby enhancing the effect of small changes in the bacterial amount.

SLIC measures laser scattering every second, measured in mV, and contextualizes measurements to the initial calibration measurements of the experimental beginning, leading to tracking of bacterial growth measured in Delta mV.

## 3. Results

### 3.1. Proof of Concept

**Figure 2:**
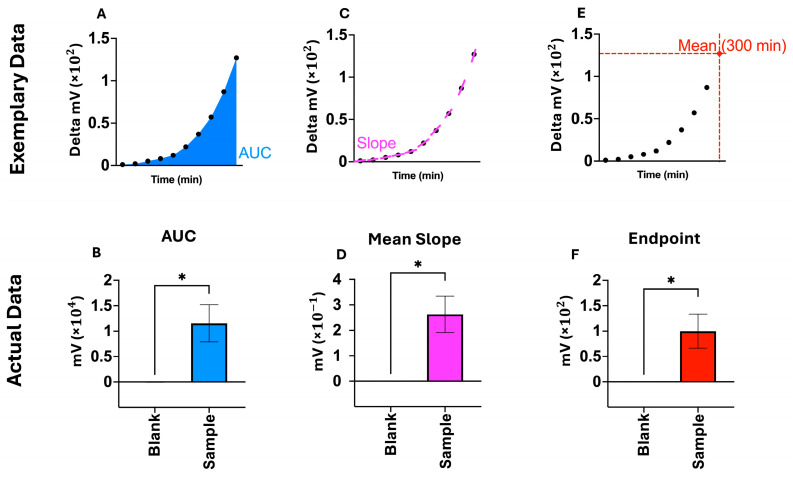
Proof of concept, demonstrating pathogen growth signals using three different statistical methods in accordance with the microbiology findings. (**A**): Area under the curve (=AUC), enclosed surface of curve, shown in blue on exemplary data panel; (**B**): Pooling of AUC-values of 3 biological replicates with positive pathogen detection in microbiological diagnostics (Smp), showing significance (unpaired *t*-test) against the mean-slopes of the 3 allocated blank curves (Blc) (*p*-value < 0.05), SEM; (**C**): Mean slope of curve across experiment, calculated using the slopes of each minute for a total duration of 300 min, shown on exemplary data panel; (**D**): Pooling of mean slopes of 3 biological replicates with positive pathogen detection in microbiological diagnostics, showing significance (unpaired *t*-test) against the mean slopes of the 3 allocated blank curves (*p*-value < 0.05), SEM; (**E**): Endpoint measurement as mean delta mV of the 300th min of experimental runtime, signifying the final y-value that was reached; (**F**): Pooling endpoints of 3 biological replicates with positive pathogen detection in microbiological diagnostics, showing significance (unpaired *t*-test) against the endpoints of the 3 allocated blank curves (*p*-value < 0.05), SEM. * *p* < 0.05.

The previously mentioned mathematical analysis tools were tested to verify if the present bacterial infection could be accurately differentiated from their blank curve. This is illustrated in Figure 2. Pooled data from the same three patients with positive microbiological pathogen (a positive microbiological culture result obtained in the Department of Microbiology of the University Hospital Bonn) results were used for each of the three tools (AUC, mean slope, and endpoint analysis) and compared to their corresponding blank curve data points (e.g., AUC of blank, mean slope of blank, etc.). For all three methods, SLIC results were deemed significant (*p*-value < 0.05), and the corresponding curves were labeled as growth, fully consistent with microbiological findings. This validation led to the subsequent use of these three methods to evaluate growth in the following experimental designs.

### 3.2. Coagulation Interference Tests

The use of coagulation-influencing agents was incorporated into the developed SOP to evaluate whether initiation or inhibition of blood clotting is necessary, considering blood contamination in most intraoperative tissue biopsy samples. Tested samples were either processed without additives (Falcon tube) as a control or in the presence of the procoagulant silicate, as well as both anticoagulants EDTA and heparin. Differently prepared samples of the same patient were tested simultaneously using SLIC (version 7.1). Five patients with positive microbiological findings were included in this comparison (Figure S1).

**Figure 3:**
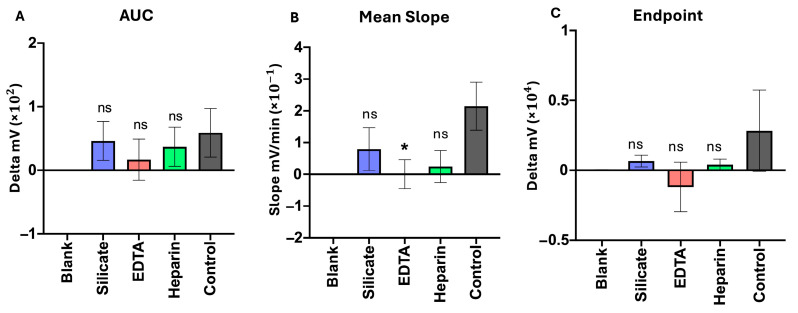
Coagulation interference tests, run on 5 biological replicates of patients with positive microbiological pathogen findings based on a five-hour SLIC run. The procoagulant silicate and both anticoagulants, EDTA and heparin, were used for this comparison, and each was tested against the use of a plain tube without agents influencing coagulation as a control. Analysis was performed using an ordinary one-way ANOVA and Dunnett’s multiple comparisons post hoc test. (**A**): Area under the curve (=AUC) values depicted via their means as bars. Comparisons are shown in relation to the control (no agents added); no significant differences were detected, *p* > 0.05, SEM; (**B**): Mean slopes within a SLIC run depicted via their means as bars. Comparisons are shown in relation to the control. EDTA shows a significant decrease in mean slope (*p* < 0.05), SEM; (**C**): Endpoint measurements depicted via their means as bars. Comparisons are shown in relation to the control; no significant differences were detected (*p* > 0.05), SEM. * *p* < 0.05; ns = not significant.

AUC analysis showed no superiority of added agents. The curve dynamics in the presence of silicate and heparin displayed high similarity to those in the absence of additives (control) (*p*-values being 0.994 and 0.957, respectively). EDTA, in contrast, showed a tendency to reduce curve-AUC, resulting in a slightly lower *p*-value of 0.710 (Figure 3).

Among the samples investigated, mean slopes reached the highest values when no additive was used (mean = 0.149 mV/min), while the addition of anti- or coagulation revealed a tendency to a decrease with significant slope reduction during usage of EDTA (*p* = 0.039). Decreasing tendencies with added coagulation additives were also observed concerning the max endpoint of the curves.

### 3.3. Storage Tests

To assess reproducibility, we created x-fold data of our results under different storage conditions relative to the original value from the fresh test for each patient. Tests were repeated after intermediate storage at 4 °C for one week, and for 2, 4, and 8 weeks at −20 °C and −80 °C.

AUC, slope, and endpoint ratios were compared to an ideal fold of 1, indicating, therefore, that storage and fresh test results are 100% identical. Deviations from this ideal fold were tested using an unpaired *t*-test for one week of storage at 4 °C and an ordinary one-way ANOVA and Dunnett’s multiple comparison post hoc analysis for the rest. Insignificant deviations were considered to confirm reproducibility.

**Figure 4:**
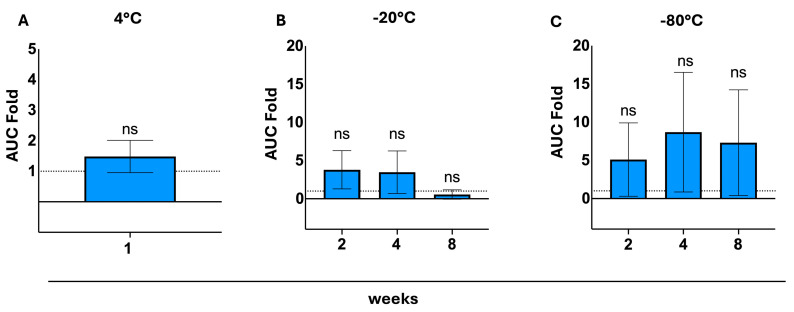
Testing reproducibility using an AUC fold; AUC ratio between fresh test and storage test was calculated and compared to a control (ideal fold of 1; equivalent to 100% similarity); (**A**): AUC-ratios after 1 week of storage at 4 °C, with no significant deviation from the control detected (via an unpaired *t*-test), *p* > 0.05, SEM; (**B**): AUC ratios after 2, 4, 8 weeks of storage at −20 °C, with no significant deviation from the control detected (via an ordinary one-way ANOVA and a Dunnett’s multiple comparisons post hoc test), *p* > 0.05, SEM; (**C**): AUC ratios after 2, 4, and 8 weeks of storage at −80 °C, with no significant deviations from the control detected (via an ordinary one-way ANOVA and a Dunnett’s multiple comparisons post hoc test), *p* > 0.05, SEM. ns = not significant.

After one week of storage at 4 °C, AUC folds (using tubes without anticoagulation for processing) show no significant deviation from the control (*p* = 0.961). Intermediate storage at −20 °C and −80 °C (for a duration of 2,4 and 8 weeks) shows a comparably bigger tendency of fold spread while not being considered significantly different from the ideal fold (*p*-values being 0.603, 0.718, and 0.998 for −20 °C, and 0.883, 0.602, and 0.771 for −80 °C, respectively) (Figure 4).

**Figure 5:**
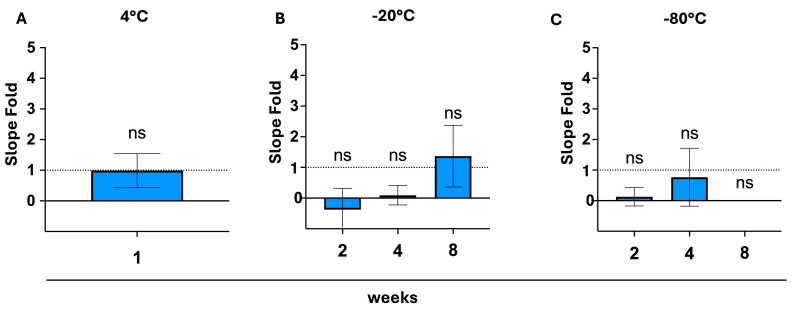
Testing reproducibility using a slope fold. The slope ratio between the fresh test and storage test was calculated and compared to the control (an ideal fold of 1 is equivalent to 100% similarity). (**A**): Slope ratios after 1 week of storage at 4 °C (blue bar), with no significant deviation from the control detected (via an unpaired *t*-test), *p* > 0.05, SEM; (**B**): Slope ratios after 2, 4, 8 weeks of storage at −20 °C tubes (blue bars), with no significant deviation from the control detected (via an ordinary one-way ANOVA and a Dunnett’s multiple comparisons post hoc test), *p* > 0.05, SEM; (**C**): Slope ratios after 2,4,8 weeks of storage at −80 °C (blue bars), with no significant deviations from the control detected (via an ordinary one-way ANOVA and a Dunnett’s multiple comparisons post hoc test), *p* > 0.05, SEM. ns = not significant.

Using the mean slope fold, results showed to be reproducible for 1 week storage at 4 °C (*p* = 0.981). Investigated storage conditions at −20 °C and −80 °C continue to show an increase in fold spread. When tested against the ideal constructed fold, results remain insignificant (*p*-values being 0.209, 0.954, and 0.613 for −20 °C, and 0.999, 0.999, and 0.177 for −80 °C, respectively) (Figure 5).

**Figure 6:**
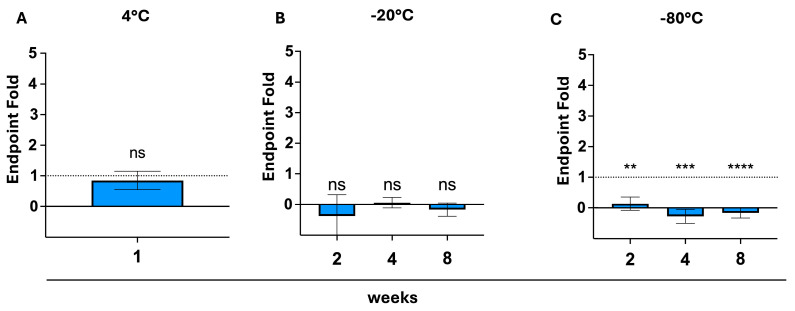
Testing reproducibility using an endpoint fold. Slope ratio between fresh test and storage test was calculated and compared to the control (an ideal fold of 1 is equivalent to 100% similarity); (**A**): Endpoint ratios after 1 week of storage at 4 °C (blue bar), with no significant deviation from the control detected (via an unpaired *t*-test), *p* > 0.05, SEM; (**B**): Endpoint ratios after 2, 4, and 8 weeks of storage at −20 °C (blue bars), with no significant deviations from the control detected (via an ordinary one-way ANOVA and a Dunnett’s multiple comparisons post hoc test), *p* > 0.05, SEM; (**C**): Endpoint ratios after 2,4, and 8 weeks of storage at −20 °C (blue bars), with significant deviations from the control detected at 2, 4 and 8 weeks of storage (via an ordinary one-way ANOVA and a Dunnett’s multiple comparisons post hoc test), *p* < 0.05, SEM. ** *p* < 0.01, *** *p* < 0.001, **** *p* < 0.0001; ns = not significant.

Endpoint ratios demonstrated similar trends in curve reproducibility. Storage at 4 °C showed close similarity to the constructed ideal fold, resulting in a non-significant deviation (*p* = 0.628). The fold shows increased spread when stored at either −20 °C or −80 °C and is still considered insignificantly different from the control for all tested durations at −20 °C (*p*-values being 0.091, 0.413, and 0.256). Storage at −80 °C led to significant growth reduction for all investigated storage durations (*p*-values being 0.002, 0.0002, and <0.0001) (Figure 6).

Thus, the storage of the sample is considered possible for a duration of 1 week at 4 °C and 2, 4, and 8 weeks at −20 °C, and hence, results can still be obtained after the tested freezing periods with reproducible results.

### 3.4. Sample Amount

To expedite the runtime and increase signal output, the tested sample volume was modified throughout the titration tests. Tested amounts included values above and below the previously used 100 µL sample. Six biological replicates were investigated, obtained from patients with positive microbiological pathogen findings. The analysis timeframe was set to 5 h as well. Results of varied sample amounts were compared to those of a 100 µL sample using an ordinary one-way ANOVA and a Dunnett’s multiple comparisons post hoc test.

Deviations from the sample quantity led to no significant results using AUC (*p*-values being 0.999, 0.999, 0.999, and 0.423, respectively). A slight increase in AUC was detected using 300 µL (Mean AUC being 642 mV for 100 µL and 13,123 mV for 300 µL, respectively).

Mean slopes increased proportionally with the tested sample amount (0.01974 mV for a 25 µL sample, 0.05354 mV for 100 µL, and 0.2217 mV for 300 µL), but remained insignificantly different from the results generated with 100 µL samples (*p*-values being 0.999, 0.999, 0.999, and 0.636, respectively).

**Figure 7:**
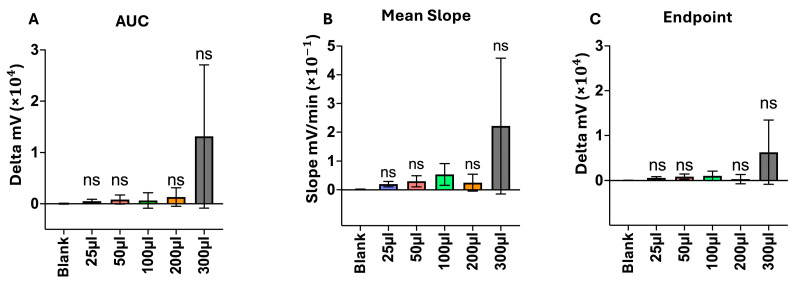
Influence of sample volume, tested on 6 biological replicates of patients with positive microbiological pathogen findings on the basis of a five-hour SLIC run. Comparisons are calculated using an ordinary one-way ANOVA and a Dunnett’s multiple comparisons post hoc test. (**A**): AUC values depicted via their means as bars. Comparisons are shown in relation to100 µL, and no significant differences were detected, *p* > 0.05, SEM, with the highest AUC reached using 300 µL of the sample; (**B**): Mean slopes depicted via their means as corresponding bars, and comparisons are shown in relation to 100 µL, with no significant differences detected, *p* > 0.05, SEM, and the highest means were reached using 300 µL of the sample volume; (**C**): Endpoint measurements depicted via their means as corresponding bars. Comparisons are shown in relation to 100 µL, and no significant differences were detected, *p* > 0.05, SEM, with the highest endpoints reached using 300 µL of the sample volume. ns = not significant.

The same applies to the endpoints of curves, with endpoint means of 5.857 mV for 25 µL, 10.43 mV for 100 µL, and 63.02 mV for 300 µL (with *p*-values being 0.999, 0.999, 0.999, and 0.607, respectively). Hence, a rise in the signal was generated through scattered light; these results were without statistical significance when compared to 100 µL samples. Figure 7 shows the respective findings below.

## 4. Discussion

Given the absence of a flawless diagnostic test for joint infections, standardized criteria are vital for both clinical diagnosis and research [24]. No single routine test can reliably diagnose all cases of PJI, each having its advantages and disadvantages [16,24,25]. A combination of clinical, serum, and synovial tests, as well as microbiological and histological examinations, can yield a diagnosis in most cases [26]. While joint aspiration or arthrocentesis is the most commonly conducted pre-operative invasive procedure to identify joint infections, tissue sample analysis proves more effective in detecting co-infections [16,17,18,27,28]. If preoperative joint aspiration is not feasible, pathogen detection and the subsequent diagnosis of joint infection become challenging [29]. Thus, periprosthetic tissue biopsies remain the primary source of microbiological samples [22].

To our knowledge, SLIC has not yet been tested on solid tissue samples, whilst previous studies have focused on in vitro settings or liquid samples such as blood culture bottles [15]. This study extends sample acquisition to solid samples while restricting itself to microbiologically verified tissue samples (verified via a culture-based pathogen identification within the microbiological department of the University Hospital Bonn). Assessing multiple steps of sample preparation, various experimental designs were necessary, leading to decreased comparability between different experimental sets. Limiting our sample collection in that regard enabled us to focus on growth maximization regarding singular treatment methods. However, this approach proposes a profound selection bias, which we plan to eliminate within further projects by incorporating samples independently of their infection status within one continuous setup of sample preparation. Of course, samples deriving from different patients underlie an invariable variation to some extent. To limit this further, we secluded the sample collection to the periprosthetic membrane and tissue of the articular capsule. To gather further knowledge of sample comparability, the tissue was weighed before usage. Since we were interested in testing SLIC for a variety of different PJI cases and their causative pathogens, we decided to refrain from narrowing our sample collection to a singular pathogen and tried to maximize SLIC's measured growth signal across different biological replicates by adjusting the methodology of sample preparation. Cuvettes within SLIC are typically filled with buffer solutions, transforming solid tissue into a liquid solution that allows for light transmission without impairing scattered signal collection remains a challenge [15]. Tissue homogenization, a well-established technique in biological research for years, is a reliable method to facilitate pathogen release and microbial load detection [30,31]. Tissue grinding has shown potential as a mechanical homogenization method. Tissue disruption was performed using vortex, a simple and accessible tissue homogenization method [21,27,31]. This allowed cost and resource-efficient reproducibility of our experimental setup.

Tissue pretreatments have been utilized and described over the past 20 years, reflecting the diversity and complexity of PJI and SA diagnosis [9,16,21,29,32,33]. Our study also showed that simple sample pretreatment can aid in elucidating pathogen growth in infected tissue samples.

Bacterial culture, a fundamental technique in microbiology, remains a widely applied technique, especially for detecting pathogens and their resistance, requiring, in some cases, over 2 weeks of incubation [29]. Time to positivity and time to treat are crucial factors in PJI and SA therapy, improving treatment efficiency and consequently patient survival [5,28,34]. While some studies show that SLIC allows tracking of pathogens and their response to various antibiotics when used with bacterial strains [15,35] (Figure S2), others indicate a lack of diagnostic accuracy with the use of blood culture bottles as clinical samples [36]. However, further research is necessary to fully understand SLIC's potential as well as its limitations for each sample entity.

Many features remain unclear, primarily because the technique is still in its initial stages and requires further development to reach its full potential. Samples derived from revision surgery usually show blood contamination to some extent, which might affect the diagnostic precision [24]. Different sampling methods have been described in the literature to propose a solution for this inevitable contamination. Lew et al. use heparin as an anticoagulant mixed in vitro with sodium, lithium, or ammonium salt to perform sample preparation, as heparin inactivates specific coagulation proteins and enzymes [25]. However, Beutler et al. reported an interference of heparin with the polymerase chain reaction (PCR), a further technique used in the diagnosis of PJI [28]. EDTA has been shown to be a stable preservation additive for synovial fluid tubes, allowing therefore a precise cell count and safe storage for up to 48 h at 4 °C [20]. These tubes may be useful to limit clot formation [27]. Lam et al. demonstrated that heparin, EDTA, and citrate are similar for DNA analysis after 6 h of venesection, with EDTA being the preferred anticoagulant for delayed blood processing of over 24 h [29]. Our findings show a clear advantage for heparin and Falcon tubes compared to EDTA tubes. This aligns with the findings of Finnegan and Percival, who demonstrated that EDTA acts as an antimicrobial and antibiofilm agent. It effectively chelates and potentiates bacterial cell walls and destabilizes biofilms by sequestering calcium, magnesium, zinc, and iron [37]. Serum tubes with silica-coated granules and polyacrylic ester gel show equivalent results to Falcon and heparin tubes.

To further validate the efficacy of our methods, we investigated the reproducibility of results after cold (4 °C) and frozen (−20 °C and −80 °C) storage. Storage testing is crucial for assessing the stability and reliability of samples over time, particularly in the context of potential delays between sample collection and analysis. Additionally, safe and reliable storage is highly relevant for research purposes. For more than 40 years, static cold storage (SCS) has dominated the ex vivo preservation of tissue allografts [32]. Silva et al. applied frozen storage at −4 °C to sonication samples for 7 days and demonstrated that a minor decrease in the bacterial load did not affect culture positivity, showing similar findings to samples processed within 2 to 4 h of removal [33]. This storage was conducted in a sealed and moisturized environment, best achieved with sterile saline or Ringer’s solution in physical containers rather than plastic bags, as also reported by Cielinski et al. [34]. Smith and Morin proved a loss of quality of dry storage of DNA samples between −20 °C and 4 °C over longer terms, contrary to −80 °C where long storage showed to be safe regardless of additives [38]. Nagy’s overview on tissue preservation methods for molecular genetic analyses showed that freezing at −20 °C is effective for short to mid-term storage. However, −80 °C and even −150 °C are superior, allowing long-term to very long-term storage of tissue samples without impairing results. These relatively simple methods, however, require an electricity or dry ice source [39]. Our findings align closely with the literature, showing reproducibility after frozen storage, as indicated by the insignificant deviations observed in the *t*-test results. This supports the reliability of our protocols after storage at −20 °C for up to 8 weeks. However, cryopreservation at −80 °C showed consistent results in 2 of our 3 statistical analyses, proving that storage for up to 8 weeks may be safe and reproducible. Further research using larger scales and testing is required to ensure the robustness of our storage approach.

Conversely, we observed higher growth at 4 °C after a storage period of 1 week compared to the same samples that were directly tested. Comparable results were reported by Van Cauter et al. regarding the culture of *Staphylococcus epidermidis*, where they showed that samples need to be cultivated as soon as possible, optimally within 2 h after sampling. Temporary storage in a refrigerator at 4 °C also positively influenced bacterial viability [40]. We, therefore, recommend intermediate storage of samples at 4 °C if direct testing of the samples is not possible.

In a study published in 2023, Williamson et al. proved that a critical aspect of tissue sampling is the sample size or quantity, where a minimum volume is necessary to reliably detect pathogens [41]. Pathogen load and distribution can significantly impact detection. While small samples may lead to false negatives, larger samples increase the likelihood of detecting the pathogen but require more processing time, making accurate detection challenging [41,42,43]. Kayser et al. stated that tissue volume, macroscopic aspect, boundary surface densities, and spatial features like shape and color allow for stratified sampling, a specific technique that improves the accurate detection of pathogens, rather than random sampling [44]. Our study tested periprosthetic tissues, synovial tissue, and joint capsules, applying, therefore, stratified sampling to maximize the chances of pathogen detection, as recommended by Li et al. [45]. An initial tissue pretreatment facilitates pathogen extraction and enhances their distribution in a buffer solution before being transferred to the SLIC cuvettes. Higher solution concentrations may impair light collection, while lower concentrations might result in false negatives after five hours of testing [15]. Our results showed similar outcomes for samples ranging from 25 µL to 300 µL, with biological replicates displaying higher thresholds for larger volumes, though without a significant difference within the 5 h test period.

Due to the multidisciplinary nature of joint infections (PJI and SA), management of these infections remains a challenge. Correct microbiological diagnosis is crucial for effective treatment and successful surgical intervention [46]. Currently, samples should still be obtained for culture to test the susceptibility of the pathogen(s) involved, while new methods to diagnose underlying infections are slowly evaluated for their usefulness. Emerging biomarkers such as alpha-defensin or TNF-alpha might be able to hint at a process of inflammation, but fail to offer adequate sensitivity or specificity to influence the diagnostic process of PJI on their own [6,47]. Non-culture techniques are a valuable supplemental tool in patients with culture-negative PJI caused by fastidious or slow-growing microorganisms and in patients who have previously been on antibiotics, allowing for earlier and more effective treatment [15,46].

Due to the diagnostic gap concerning periprosthetic joint infection, multiple new methods have arisen to add valuable insights concerning the likelihood of underlying infection, such as the microcalorimetry of synovial fluid [48] or the assessment of synovial fluid viscosity [49]. Additional approaches for lower bacterial detection limits are tested outside of the context of joint infections to reduce bacterial detection limits, such as the usage of acoustic aptasensors for *S. aureus* detection within dairy products [50]. Whatever their origin, such procedures still have to prove themselves against already-established methodology in microbiological departments like the polymerase chain reaction (PCR) [51,52].

While being widely recognized, they often come with a profound cost for equipment and infrastructure. Since PJI prevalence is rising within the context of demographic change, cost efficiency will continue to gain relevance. SLIC embodies such a proposal for cost-efficient and less time-consuming diagnostic tools. Our current research evaluates the methodology of sample preparation to increase depiction of bacterial growth within known present infections. Future research could apply its results as a standardized sample treatment and evaluate its sensitivity and specificity, as well as its level of detection within a higher number of biological replicates. Additional focus could go towards assessing the efficiency of bacterial extraction as a form of quality control. Should it render reliable testing results, SLIC could pose as a novel approach to aid in the diagnosis of joint infections.

## Data Availability

The original contributions presented in the study are included in the article/Supplementary Materials. Further inquiries can be directed to the corresponding author.

## Supplementary Materials

The following supporting information can be downloaded at https://www.mdpi.com/article/10.3390/bios15120795/s1. Figure S1: Coagulation interference tests. Figure S2: SLIC run of bacterial culture with and without antibiotics (using an *S. aureus* lab strain and an *S. haemolyticus* tissue sample strain).
