# Supplementary material for: A Novel Approach for Tissue Analysis in Joint Infections Using the Scattered Light Integrating Collector (SLIC)
Source: Biosensors (Basel). 2025 Dec 4;15(12):795. doi: 10.3390/bios15120795 (PMC12731015; doi:10.3390/bios15120795)
Supplement: Supplementary file 1 [file biosensors-15-00795-s001.zip › biosensors-3937118-supplementary.pdf]

Supplementary Figures

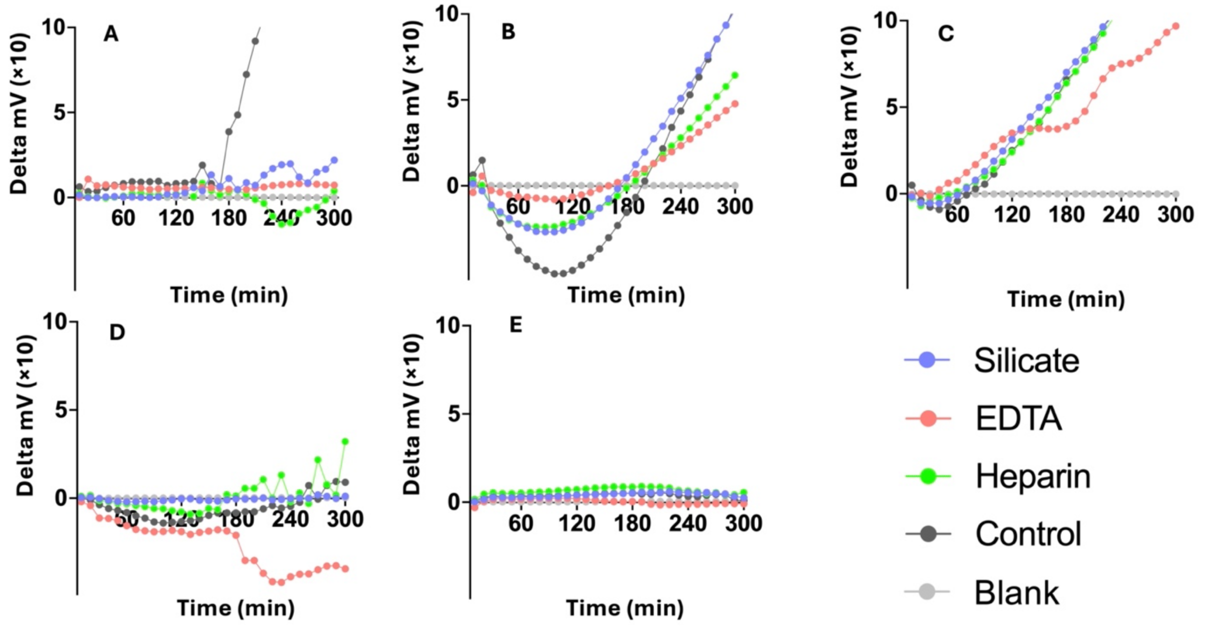

Figure S1. Coagulation interference tests, run on 5 biological replicates of patients with postitive microbiological pathogen-findings (A-E) on the basis of a five-hours SLIC-run, tubes prepped with silicate (procoagulant), EDTA (anticoagulant 1) or heparin (anticoagulant 2) were used for this comparison and tested against the use of a plain tube without anticoagulation agents (= control). Data is depicted in mV output in relation to time measured in minutes. Each patient is represented via one graph summarizing data points of added agents and a control.

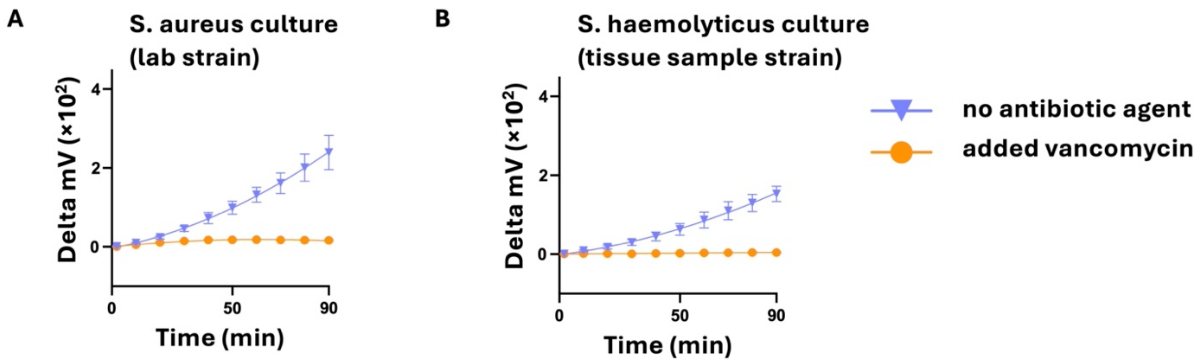

Figure S2. SLIC-run of bacterial culture with and without antibiotics (using an S.aureus lab strain and a S. haemolyticus tissue sample strain). SLIC output [Delta mV] of bacteria without antibiotic agent (shown in purple) and with addition of vancomycin (shown in orange) as antibiotic agent, showing a reduction of growth on three technical replicates.
